# Loss-of-function of the hippo transducer TAZ reduces mammary tumor growth through a myeloid-derived suppressor cell-dependent mechanism

**DOI:** 10.1038/s41417-022-00502-0

**Published:** 2022-07-15

**Authors:** He Shen, Yuwen Zhang, Elliot D. Kramer, Eriko Katsuta, Yin Wan, Yanmin Chen, Jianmin Wang, Yali Zhang, Junko Matsuzaki, Costa Frangou, Scott I. Abrams, Jianmin Zhang

**Affiliations:** 1 Department of Cancer Genetics & Genomics, Roswell Park Comprehensive Cancer Center, Elm and Carlton Streets, Buffalo, NY 14263 USA; 2 Department of Translational Immuno-Oncology, Roswell Park Comprehensive Cancer Center, Elm and Carlton Streets, Buffalo, NY 14263 USA; 3 Department of Immunology, Roswell Park Comprehensive Cancer Center, Elm and Carlton Streets, Buffalo, NY 14263 USA; 4 Department of Cell Stress Biology, Roswell Park Comprehensive Cancer Center, Elm and Carlton Streets, Buffalo, NY 14263 USA; 5 Department of Molecular & Cellular Biology, Roswell Park Comprehensive Cancer Center, Elm and Carlton Streets, Buffalo, NY 14263 USA; 6 Department of Biostatistics and Bioinformatics, Roswell Park Comprehensive Cancer Center, Elm and Carlton Streets, Buffalo, NY 14263 USA

**Keywords:** Tumour immunology, Cancer genetics

## Abstract

TAZ, one of the key effectors in the Hippo pathway, is often dysregulated in breast cancer, leading to cancer stemness, survival, and metastasis. However, the mechanistic bases of these tumor outcomes are incompletely understood and even less is known about the potential role played by the non-malignant cellular constituents of the tumor microenvironment (TME). Here, we revealed an inverse correlation between TAZ expression and survival in triple-negative breast cancer (TNBC), but not other subtypes of breast cancer. We found that TAZ knockdown in two murine TNBC tumor cell line models significantly inhibited tumor growth and metastasis in immune competent but not immune deficient hosts. RNA-seq analyses identified substantial alterations in immune components in TAZ knockdown tumors. Using mass cytometry analysis, we found that TAZ-deficiency altered the immune landscape of the TME leading to significant reductions in immune suppressive populations, namely myeloid-derived suppressor cells (MDSCs) and macrophages accompanied by elevated CD8^+^ T cell/myeloid cell ratios. Mechanistic studies demonstrated that TAZ-mediated tumor growth was MDSC-dependent in that MDSC depletion led to reduced tumor growth in control, but not TAZ-knockdown tumor cells. Altogether, we identified a novel non-cancer cell-autonomous mechanism by which tumor-intrinsic TAZ expression aids tumor progression. Thus, our findings advance an understanding of the crosstalk between tumor-derived TAZ expression and the immune contexture within the TME, which may lead to new therapeutic interventions for TNBC or other TAZ-driven cancers.

## Introduction

The TME is now recognized as an integral determinant in modulating neoplastic progression, including TNBC [1]. The TME contains a variety of non-cancer cells, such as cancer-associated fibroblasts (CAFs), vascular endothelial cells, and immune cells, including T cells, B cells, neutrophils, macrophages, and myeloid-derived suppressor cells (MDSCs) [2]. Evidence indicates that these populations contribute to neoplastic progression via signals that stimulate proliferation, reduce tumor cell death, aid angiogenesis, invasion and metastasis, and increase resistance to chemotherapy, radiotherapy, and immunotherapy [3]. Indeed, newly developed immune checkpoint inhibitors (ICIs), which target negative regulatory pathways in T cells to enhance the antitumor immune responses, have led to important clinical advances and provide a new weapon against cancer [4]. Therefore, understanding how these different non-cancerous cell types contribute to tumor progression will help inspire the development of novel therapies.

Dysregulation of the Hippo signaling effector proteins YAP/TAZ is associated with tumor progression across multiple cancer types [5]. YAP/TAZ are transcriptional co-activators that interact mainly with the TEA domain (TEAD) transcription factor family of proteins and drive cell proliferation, migration, and survival [6]. High expression and nuclear localization of YAP/TAZ has been observed in breast, liver, lung, and colon cancers [5]. We previously demonstrated in breast cancer models that activation of YAP/TAZ induced epithelial-to-mesenchymal transition (EMT), resistance to apoptosis, and growth factor-independent cell proliferation through activation of EGFR signaling [7, 8]. Therefore, TAZ confers breast cancer with stem cell-like traits [9, 10]. We also recently showed that TAZ is not only a driver of basal-like breast cancer progression, but also a requirement for tumor maintenance and the establishment of metastases [11]. Thus, tumor-intrinsic TAZ expression can affect tumor progression through multiple mechanisms; however, it is not completely understood how TAZ expression impacts the immune elements of the TME, which are now regarded as major determinants governing overall tumor outcome.

Here, to assess the regulatory role of TAZ in the immune-TME, we deleted TAZ using CRISPR-Cas9 or shRNA systems in two distinct mammary tumor models of TNBC: 4T1 and EMT6 cells. We showed that the loss of TAZ significantly inhibited tumor growth and metastasis in immune competent mice. In contrast, TAZ deficiency had no significant bearing on tumor growth in immune deficient mice, highlighting the importance of the immune compartment in mediating tumor control. RNA-seq analysis of control and TAZ knockdown 4T1 tumors identified substantial alterations in immune components, indicating that TAZ expression in these tumors played an integral role in immune cell mobilization to the TME. Using mass cytometry analysis, we demonstrated significant alterations in immune cell subsets within the TME of TAZ-deficient tumors of immune competent hosts, namely leading to reductions in MDSCs and macrophages. Furthermore, depletion of MDSCs reduced tumor growth in TAZ-expressing control, but not TAZ-deficient tumors, suggesting that tumor TAZ expression acts through MDSC-dependent mechanisms. Altogether, we uncovered a novel non-cancer cell-autonomous mechanism by which tumor intrinsic TAZ expression aids tumor progression in the field of TNBC.

## Methods

### Cell lines and cell culture

4T1 cells were purchased from ATCC. E0771 cells were purchase from CH3 Biosystems. EMT6 cells were kindly provided by Dr. Yurij Ionov and MB49 cells were kindly provided by Dr. Yuesheng Zhang both at the Roswell Park Comprehensive Cancer Center. 4T1, EMT6, E0771, and MB49 cells were cultured in DMEM (Corning, NY) supplemented with 10% fetal bovine serum and 100IU Penicillin & 100 µg/ml Streptomycin. All cells were cultured in a humidified atmosphere of 95% air and 5% CO_2_ at 37 °C. All cell lines were confirmed mycoplasma-free.

### Plasmids, shTaz and sgTaz lentiviral production

shNT (SHC002) and shTaz (TRCN0000095952 & TRCN0000095949) constructs were purchased from Sigma-Aldrich (St. Louis, MO). sgcon, sgTaz (Target sequence: GAGGATTAGGATGCGTC-AAG) and Cas9 expression constructs were a gift from Cellecta (Mountain View, CA) Briefly, for lentiviral packaging, shRNA plasmid, Δ8.9 and Vsvg were co-transfected into 293 T cells with the tremeGENE 9 DNA Transfection Reagent from MilliporeSigma (Burlington, MA). Viral supernatants were collected on days 3 and 4 after transfection. Cas9, sgControl or sgTaz plasmids, psPAX2 and pMD2.G were co-transfected into 293 T cells with the tremeGENE 9 DNA Transfection Reagent. Viral supernatants were collected on days 3 and 4 after plasmid transfection.

### In vivo tumor growth

1 × 10^5^ sgControl or sgTaz 4T1 or 5 × 10^5^ sgControl or sgTaz EMT6 cells were injected into the 4^th^ mammary fat pad of 6–8-week-old female SCID or BALB/c mice. The SCID mice were bred at Roswell Park. The BALB/c mice were purchased from Charles River Laboratories (Catskill, NY). Tumor sizes were measured once a week using a digital caliper. Tumor growth was also detected by the In Vivo Luminescence Imaging System. All animal studies were approved by the Institutional Animal Care and Use Committee of Roswell Park.

### Tissue dissociation

The primary tumor masses were surgically removed following euthanasia. Tumors were transferred to gentle MACS C-tubes from Miltenyi Biotec (Waltham, MA) in the presence of 2 mL of a 1X collagenase/hyaluronidase cocktail from Stemcell Technologies (Cambridge, MA) and dissociated using the gentleMACS™ Dissociator per manufacturer’s instructions. The suspension was further incubated at 37 ˚C in a rotating incubator and then strained through 100 μm SureStrain from Laboratory Products Sales (Rochester, NY) filters prior to resuspension in PBS.

### Flow cytometry analysis

Isolated cells were subjected to ACK lysis to remove RBCs. One million cells were suspended in staining buffer (PBS + 0.5% BSA + 2 mM EDTA) and treated with Mouse Fc block from (BD Biosciences; NJ) followed by incubation with the conjugated primary antibodies listed in Table S3, or in the case of the Gr-1 analysis, stained separately with the anti-CD45 and anti-CD11b antibodies listed in Table S3 along with PE-Cy5 anti-Gr-1 (Biolegend, #108409). Immunostained samples were then incubated with DAPI (Thermo-Fisher Scientific; NY) for dead cell exclusion. Samples were acquired on the LSR II flow cytometer (BD Biosciences) using FACSDiva version 6.1.3 software. Data analysis was performed using FCS Express 7.0.

### Immunoblot analysis

For immunoblot analysis, cells were lysed in RIPA buffer (Boston Bio-Products; MA) in the presence of protease and phosphatase inhibitors (Thermo-Fisher Scientific). Protein concentration was determined using the Bradford protein assay. Briefly, BSA standards at varying concentrations were made to create a standard curve. Standards were made using RIPA buffer. Absorbance was read at 650 nm and protein concentrations were calculated based on the slope of the standard curve. 20–30 ug of protein was loaded, separated by SDS-PAGE, and then transferred onto PVDF membranes (EMD Millipore). Membranes were blocked in 5% milk in the Tris Buffered Saline with Tween (TBST) for 1 h and incubated overnight at 4 °C with the primary antibodies. The next day membranes were incubated with anti-mouse or anti-rabbit secondary antibody (Bio-Rad). Proteins were detected using Peirce ECL western blotting substrate. Anti-TAZ antibody (#83669) was purchased from Cell Signaling Technologies (Danvers, MA) and anti-GAPDH antibody (Y1041) was purchase from Ubiquitin-Proteasome Biotechnologies (Dallas, TX).

### Colony formation assay

For colony formation experiments, 200 cells were plated in a 6-well plate allowing growth to occur for 7–10 days. The plates were washed once with PBS, fixed with 4% paraformaldehyde for 10 min, and stained with crystal violet for 30 min. Colony numbers were counted under the microscope.

### Mass cytometry assay and data analysis

A single cell suspension for each sample was analyzed by Helios (Fluidigm; CA). FCS files were then normalized, as described [12]. Nucleated single cells were manually gated by DNA intercalators 191Ir/193Ir and event length. Dead cells were excluded by Cisplatin 194Pt/195Pt staining. Live single cells were manually gated, and corresponding FCS files were exported for data analyses in Cytobank and Flowjo. For viSNE analysis in Cytobank, typically 15,000 to 30,000 live singlet events/sample were utilized to generate t-SNE maps with 2000 or 3000 iterations, perplexity at 30 or 60, and theta value at 0.5. Equal events were used for each batch of viSNE analysis, which was repeated at three time with a setting change.

### RNA-seq, NanoString immunology panel analysis and RT-qPCR

For the RNA-seq analysis, total RNA was extracted from sgControl or sgTAZ 4T1-generated whole-tumors using Trizol Reagent (Thermo-Fisher Scientific) according to the manufacturer’s instructions. The RNA samples were subjected to transcriptome sequencing (RNA-seq) with an Illumina HiSeq 2000 sequencer in the Roswell Park Genomic Shared Resource. Raw reads that passed quality filter from Illumina RTA were mapped to the mm10 mouse reference genomes and corresponding GENCODE (v12) annotation databases using STAR two-pass algorithm [13]. The mapped bam files were further QCed using RSeQC [14], a quality control Bioconductor R package for RNA-seq data, to identify potential RNA-seq library preparation problems. From the mapping results, the read counts for genes were obtained by featureCounts from Subread [15]. Transcript level quantification were generated using kallisto [16], an alignment free tool. Dara normalization and differential expression analysis were preformed using DESeq2 [17], a variance-analysis package developed to infer the statically significant difference in the RNA-seq data. Pathway analysis was performed by GSEA [18] pre-ranked mode using a ranked gene list based on test statistics from differential gene expression analysis against the hallmark (H) and the canonical pathways in MSigDB. The volcano plots were generated using the Enhanced Volcano Bioconductor package and the heatmaps were generated using the heatmap R package.

For the NanoString immunology panel analysis, total RNA was extracted from sgControl or sgTAZ 4T1 cells using Trizol Reagent according to the manufacturer’s instructions. The RNA samples were subjected to NanoString immunology panel run in the Roswell Park Genomic Shared Resource.

For RT-qPCR, total RNA was harvested was extracted from sgControl or sgTAZ 4T1 cells using Trizol Reagent according to the manufacturer’s instructions. cDNA synthesis and quantitative real-time PCR were then performed. GAPDH was used as the internal control. The primer sequences were as follows:

Gapdh-F: 5’-AAC AGC AAC TCC CAC TCT TC-3’

Gapdh-R: 5’-CCT GTT GCT GTA GCC GTA TT-3’

Il33-F: 5’-TCC ACG GGA TTC TAG GAA GA-3’

Il33-R: 5’-GAG GCA GGA GAC TGT GTT AAA-3’

Tgf-β1-F: 5’-GGG CTT AGT GTT CTG GGA AA-3’

Tgf-β1-R: 5’-CCG ATG GAT CAG AAG GTA CAA G-3’

Ccl5-F: 5’-CCA ATC TTG CAG TCG TGT TTG-3’

Ccl5-R: 5’-ACC CTC TAT CCT AGC TCA TCT C-3’

Il1a-F: 5’-GAA GAA GAG ACG GCT GAG TTT-3’

Il1a-R: 5’-TCA CTC TGG TAG GTG TAA GGT-3’

Cx3cl1-F: 5’-GCT TTG CTC ATC CGC TAT CA-3’

Cx3Cl1-R: 5’-GTC TTG GAC CCA TTT CTC CTT C-3’

### Data acquisition and preprocessing from TCGA

There were 1093 breast cancer patients with clinical and primary tumor mRNA expression data from RNA sequence studies available through The Cancer Genome Atlas (TCGA). The clinical and the gene expression quantification data (mRNA expression Z-score from RNA sequence) were downloaded through the cBioportal (http://cbioportal.org) [19, 20]. PAM50 classification data was downloaded through UCSC Xena (https://xena.ucsc.edu/) [21]. TAZ target score was calculated using 22 target gene expressions as previously described [22]. Patients were classified as either target score high or low using higher quantile cutoff (25% high and 75% low). The prognostic differences were analyzed using Kaplan-Meier methods with Log-rank test. All TCGA statistical analyses were performed using R software (http:///www.r-project.org/) together with Bioconductor (http://bioconductor.org/).

### Statistical analysis

Statistical analysis was performed using GraphPad Prism software version 9.0. All data are representative of three independent experiments unless otherwise specified. P-values were determined using two-tailed Student’s t-tests (*p < 0.05, **p < 0.01, ***p < 0.001).

## Results

### TAZ is highly expressed in TNBC and correlates with poor outcome in TNBC patients

**Fig. 1:**
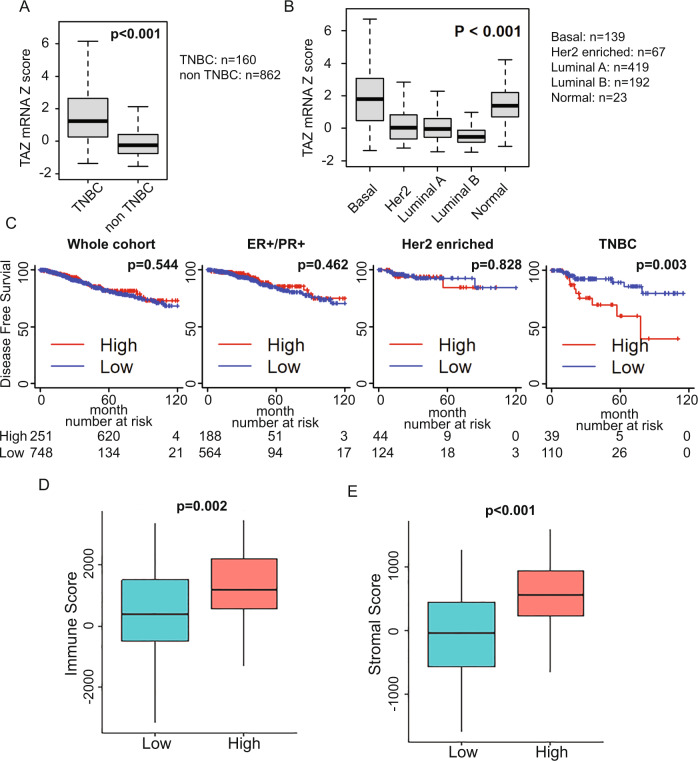
TAZ is highly expressed and correlated with poor outcome of TNBCs. **A** Comparison of TAZ mRNA expression between triple-negative breast cancer (TNBC) and non-TNBC in TCGA breast cancer datasets. TNBC; *n* = 160, non-TNBC; *n* = 862. **B** TAZ mRNA expression comparison by PAM50 classification in TCGA breast cancer datasets. Basal-like (Basal); *n* = 139, Her2; *n* = 67, Luminal A; *n* = 419, Luminal B; *n* = 192, normal-like (normal); *n* = 23. **C** Breast cancer disease-free survival Kaplan-Meier curves were generated using a TAZ target score in the whole cohort or each subtype of the TCGA breast cancer cohort. High TAZ target expression was significantly associated with high immune scores (**D**) and high stromal scores (**E**).

To determine the potential merit of TAZ expression in human breast cancer, we compared TAZ expression among different breast cancer subtypes using publicly available cohorts from the TCGA database. We found that TAZ is highly expressed in TNBC patients compared to other breast cancer subtypes (Fig. 1A). Consistently, high expression of TAZ is found in the basal-like subtype based on the PAM50 classification, which accounts for most of TNBC (Fig. 1B). To determine the impact of TAZ expression on patient survival in this TCGA dataset, we used the YAP/TAZ transcriptional target signature of 22 genes [23] as a target score to determine clinical outcome. There was no survival difference between high and low TAZ target score in the whole breast cancer cohort (Fig. 1C). Interestingly, when we stratified patients by subtype, high target score patients showed worse disease-free survival in TNBC (*P* = 0.003), whereas no differences were observed in hormone receptor-positive (ER + /PR + ) or HER2-positive subtypes (Fig. 1C).

Our data demonstrate that TAZ expression and activation are correlated with worse prognosis for TNBC patients. However, whether TAZ expression contributes to TNBC tumorigenesis, progression, or metastasis through alterations in the stromal content of the TME are largely unknown. To determine the correlation of TAZ activation and non-malignant constituents of the TME in TNBC, we further analyzed TAZ target expression and immune cell signatures utilizing the Estimation of STromal and Immune cells in MAlignant Tumor tissues using Expression (ESTIMATE) data analysis method [24]. We found high TAZ target expression was significantly associated with high immune and stromal scores (Fig. 1D, E), suggesting that TAZ expression influences the nature of the TME. To study the potential implications of this dynamic interaction between TAZ and the TME, we then developed mouse TNBC models expressing or lacking TAZ expression.

### TAZ deficiency reduces mammary tumor growth and lung metastasis

**Fig. 2:**
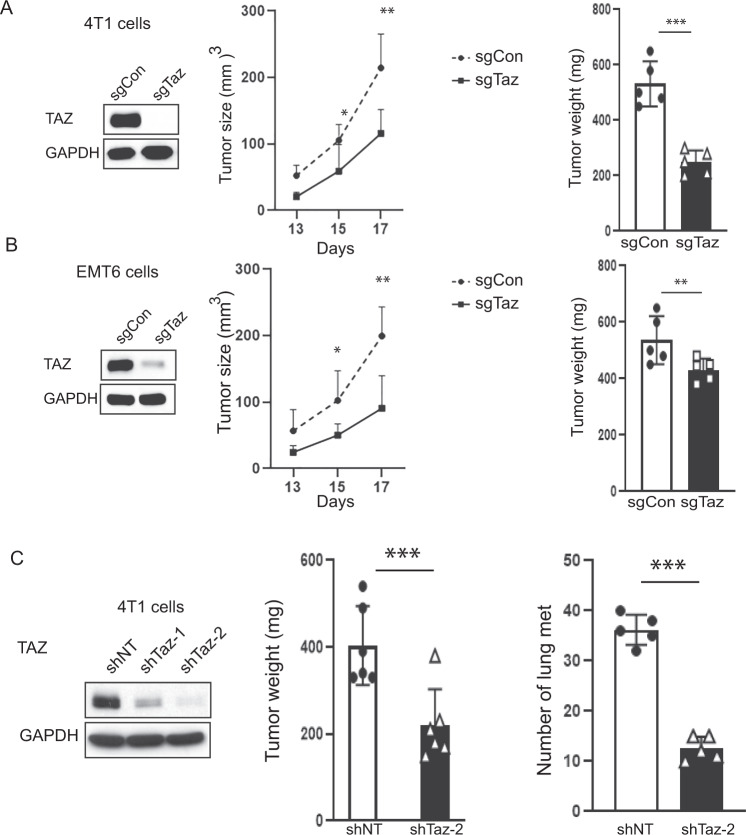
Knockdown of Taz inhibits tumor growth. **A** Immunoblotting detection of Taz knockdown in 4T1 cells; tumor growth in BALB/c mice as measured by caliper and tumor weights. GAPDH was used as a loading control. Data are shown as the mean ± SD. Unpaired two-tailed student t-test: *p < 0.05; ***p < 0.001, *n* = 6. **B** Immunoblotting detection of Taz knockdown in EMT6 cells; tumor growth measured in BALB/c as in panel **A**. GAPDH was used as a loading control. Data are shown as the mean ± SD. Unpaired two-tailed student t-test: *p < 0.05; **p < 0.01. *n* = 6. **C** Immunoblotting detection of Taz knockdown using shNT (Non-target) and two sequences independent shTaz in 4T1 cells; Tumor weights and quantification of lung tumor metastasis were measured for 4T1 shNT and shTaz 4T1 cells from BALB/c mice. Data are shown as the mean ± SD. Unpaired two-tailed student t-test: ***p < 0.001. met = metastasis nodules *n* = 6.

To determine the impact of TAZ expression on mammary tumor growth, we deleted TAZ in the TNBC cell lines 4T1 and EMT6 using the CRISPR-Cas9 system (Fig. 2A, B). TAZ deficiency significantly inhibited orthotopic mammary tumor growth in syngeneic immunocompetent mice compared to sgControl (sgCon) 4T1 or EMT6 cells (Fig. 2A, B). We did not observe overt effects of TAZ deficiency on cell proliferation in vitro, as measured using colony formation assays (Fig. S1A, B). We recently showed that TAZ expression can be important for cell survival in some human breast tumor cell lines, such MDA-MB-231 and MDA-MB-468, but not others, such as MCF7 and T47D, using TAZ knockdown approaches [11]. To extend this finding, we knocked down TAZ expression using the CRISPR-Cas9 system in the murine C57BL/6-derived TNBC model, E0771, as well as an unrelated C57BL/6-derived murine bladder tumor cell line, MB49 using shRNA system and found that TAZ knockdown in those cell line models had no adverse effects on cell growth in vitro (Fig. S1C–F). These data further support the notion that TAZ biology is complex and not necessarily mouse strain- or tumor type-dependent. Furthermore, the effect on tumor growth was lost in immune deficient (SCID) mice, which lack a functional adaptive immune system (Fig. S2A, B).

To strengthen our findings of the relationship between TAZ expression and tumor growth, we silenced TAZ in 4T1 cells using two independent TAZ-shRNA constructs (Fig. 2C). The results showed that knockdown of TAZ did not alter cell proliferation in vitro (Fig. S2C) or tumor growth in vivo in SCID mice (Fig. S2D), consistent with our prior results (Fig. S1). However, knocking down TAZ significantly inhibited tumor growth in syngeneic immune competent mice (Fig. 2C). Furthermore, we found that knocking down TAZ significantly reduced tumor metastasis to the lung (Figs. 2C & S2E). Altogether, these data revealed that a reduction in TAZ expression in two independent TNBC models using two different molecular approaches to alter TAZ expression inhibited tumor growth in syngeneic immune competent mice but not immune deficient mice. In these models, these data indicated an important role for adaptive immunity in the regulation of TAZ-mediated tumor growth and metastasis.

### RNA-seq analyses revealed TAZ knockdown alters the TME

**Fig. 3:**
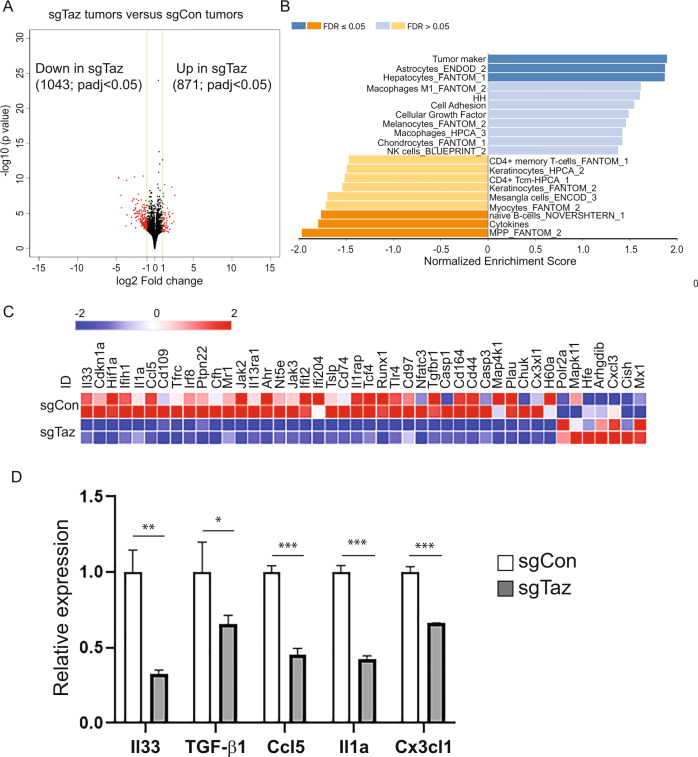
Knockdown of Taz alters the immune cell components in TME. **A** Volcano plot shows significant gene expression alterations between sgTaz and sgCon 4T1 tumors. **B** Over-representation analysis (ORA) using pathway annotations. **C** Representative heatmap data of NanoString immunology panel analyses in sgCon and sgTaz 4T1 cells. **D** qRT-PCR analyses of Il33, TGF-β1, Ccl5, Il1a, and Cx3cl1 expression in sgCon and sgTaz 4T1 cells. Relative expression was normalized by GAPDH expression. Unpaired two-tailed student t-test: *p < 0.05; **p < 0.01; ***p < 0.001.

To determine the impact of TAZ expression on the tumor cells as well as the immune TME landscape, we performed a RNA-seq study in sgCon or sgTAZ 4T1 tumors and an over-representation analysis (ORA) using pathway annotations and GO term datasets [25]. We identified 871 significantly upregulated and 1043 downregulated genes (p-adj < 0.05; Fig. 3A; Table S1). sgCon tumors were enriched for regulatory and growth processes, including tumor marker, cell adhesion, and cellular growth factor expression, as well as macrophages and NK cells. sgTAZ tumors were depleted of CD4^+^ memory T-cells, naïve B cells, and cytokines. Tumor cell-secreted chemokines have been indicated to play critical roles in inflammation, immune surveillance, and tumor progression [26]. Indeed, the NanoString immunology panel and RT-qPCR analyses found significant reductions in the expression of IL-33, TGF-β1, CCL5, IL-1α, and CX3 CL1 in sgTaz tumors compared to sgCon tumors (Fig. 3C, D). Hence, our results indicated that TAZ knockdown not only altered tumor growth processes, but also the infiltration of various immune cell components in the 4T1 tumor model.

### Myeloid populations are reduced in the TME of TAZ-deficient tumor-bearing hosts

High-dimensional mass cytometry (CyTOF) can be used to simultaneously evaluate numerous immune cell markers, and has allowed for the detailed description and quantification of tumor-infiltrating immune cells [27]. To further determine the effects of TAZ expression on altering the immune contexture of the TME, we developed a panel of 21 metal-labeled monoclonal antibodies (mAbs) for the high-dimensional analysis of multiple immune cell types using CyTOF technology, including CD3^+^CD4^+^ T cells, CD3^+^CD8^+^ T cells, B220 + B cells, MDSCs, macrophages, neutrophils, monocytes, and dendritic cells (DCs) (Table S2). Using this panel, we then performed a mass cytometry analysis in the sgCon and sgTaz 4T1 tumors. To visualize the cellular heterogeneity of the immune cells, we utilized t-SNE analysis [28]. We found similar numbers of CD45^+^ cells, as well as CD4^+^ and CD8^+^ T cells between sgCon and sgTaz 4T1 tumors (Fig. S3A, B). Consistent with the RNA-seq analyses, we observed a significant decrease in the B cells (B220^+^) in the sgTaz tumors compared to the sgCon tumors (Fig. S3A, B).

**Fig. 4:**
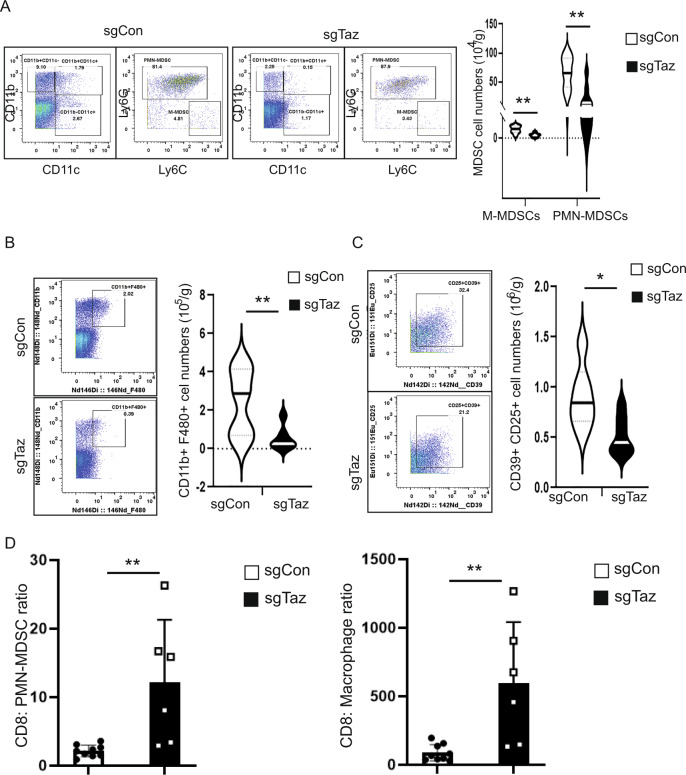
Accumulation of immune suppressive cells in 4T1 sgCon or sgTaz tumors. **A** Representative dot plots and quantification of PMN-MDSCs and M-MDSCs in the 4T1 sgCon or sgTaz primary tumors. Data are shown as the mean ± SD. Unpaired two-tailed student t-test: **p < 0.01; ***p < 0.001. **B** Representative dot plots and quantification of CD11b^+^F4/80^+^ cells in the 4T1 sgCon or sgTaz primary tumors. Data are shown as the mean ± SD. Unpaired two-tailed student t-test: **p < 0.01. **C** Representative dot plots and quantification of CD4^+^ T regulatory cells (CD25^+^CD39^+^) in the 4T1 sgCon or sgTaz tumors. Data are shown as the mean ± SD. Unpaired two-tailed student t-test: *p < 0.05; ***p < 0.001. **D** Ratio of CD8^+^ T cells with PMN-MDSCs and macrophages in 4T1 sgCon or sgTaz 4T1 tumors. Data are shown as the mean ± SD. Unpaired two-tailed student t-test: **p < 0.01.

MDSCs are a heterogeneous population of largely immature myeloid cells that have potent immune suppressive activity [29, 30]. Two major MDSC subsets have been described in mice: monocytic (M-MDSCs) and polymorphonuclear (PMN-MDSCs) [31]. Both subsets express the myeloid lineage marker CD11b and the granulocytic marker Gr-1 (which has two isoforms: Ly6C and Ly6G). PMN-MDSCs are further defined as CD11b^+^Ly6C^lo^Ly6G^+^, whereas M-MDSCs are further defined as CD11b^+^Ly6C^hi^Ly6G^−^ [32]. Interestingly, we found significant reductions in both PMN-MDSCs and M-MDSCs in sgTaz tumors compared to the sgCon tumors (Fig. 4A). We also detected significant decreases in macrophages (CD11b^+^F4/80^+^) and Tregs (CD4^+^CD25^+^CD39^+^) in sgTaz tumors compared to the sgCon 4T1 tumors (Fig. 4B, C). Although we did not observe significant changes in the absolute number of CD8^+^ T cells, we found that the ratio of CD8^+^ T cells to PMN-MDSCs or macrophages was significantly higher in sgTaz tumors compared to the sgCon 4T1 tumors (Fig. 4D), suggesting a more immune-activating TME in sgTAZ tumors. Overall, the mass cytometry analysis revealed a significant reduction in the accumulation of several immune suppressive cell types under conditions of TAZ knockdown, including MDSCs, macrophages, and Treg cells, which was accompanied by an increased ratio of CD8^+^ T cells to PMN-MDSCs or macrophages. This is consistent with a reduction in the immune suppressive nature of the TME. These findings indicated that tumor-intrinsic TAZ expression played an important role in the recruitment and accumulation of immune suppressive cells within the TME.

### MDSC depletion reduces tumor growth

**Fig. 5:**
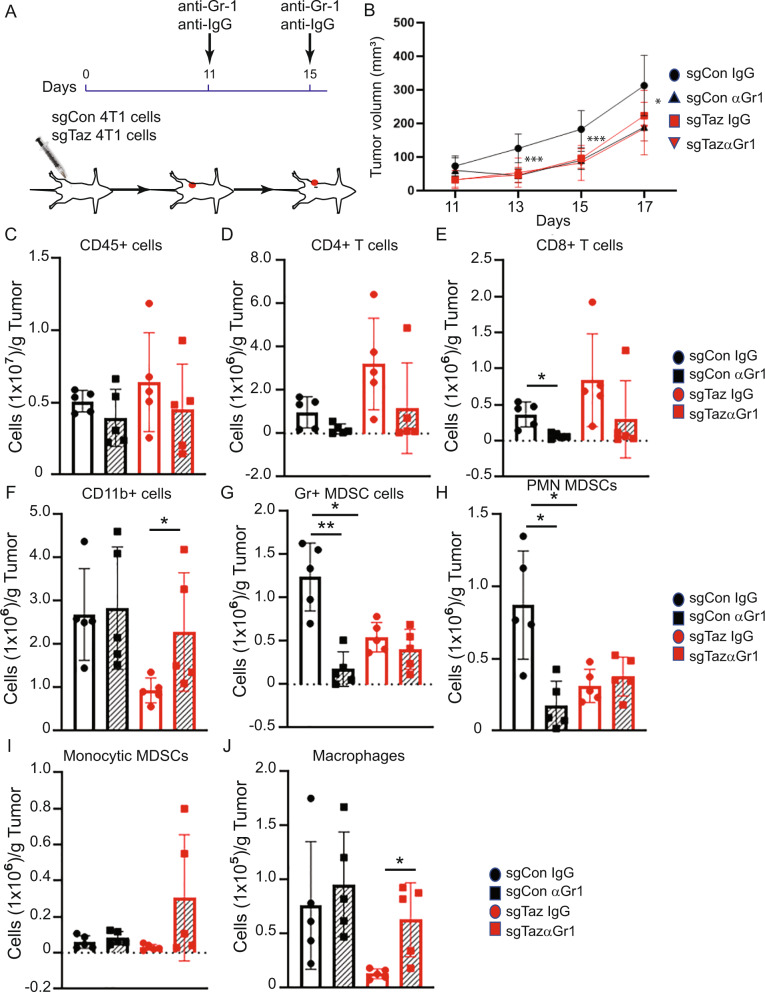
Anti-Gr-1 antibody treatment reduces tumor growth in 4T1 sgCon, but not sgTaz tumors. **A** Schematic of anti-Gr-1 antibody or isotype control antibody treatment in 4T1 sgCon or sgTaz tumor-bearing mice. **B** Tumor growth was measured by caliper after implantation of 4T1 sgCon or sgTaz 4T1 cells, with or without the indicated antibody (**A**). Data are shown as the mean ± SD. Unpaired two-tailed student t-test: *p < 0.05; ***p < 0.001. *n* = 6. **C** Quantification of CD45^+^ cells. **D** CD4^+^ T cells. **E** CD8^+^ T cells. **F** CD11b^+^ cells. **G** Gr-1^+^ MDSCs. **H** PMN-MDSCs. **I** M-MDSCs. **J** Macrophages in the 4T1 sgCon or sgTaz tumors. Data are shown as the mean ± SD. Unpaired two-tailed student t-test: *p < 0.05; **p < 0.01.

The results of the RNA-seq and mass cytometry analyses suggested that alterations in immune suppressive cells, such as MDSCs and macrophages within the TME may be responsible in part for the observed changes in tumor growth. To determine a causal role of MDSCs in mediating the growth of sgCon or sgTaz 4T1 tumors, the corresponding tumor-bearing mice were treated with anti-mouse-Gr-1 antibody or an isotype control (Fig. 5A) – this is a strategy used to deplete both subsets of MDSCs [33]. The anti-Gr-1 antibody treatment significantly reduced the growth of the sgCon tumors but not of the sgTaz tumors during this time course (Fig. 5B), suggesting that tumor-intrinsic TAZ expression acted at least in part through MDSC dependent mechanisms.

Flow cytometry analysis was performed to analyze the effect of anti-Gr-1 antibody on the immune populations within the TME. Consistent with our mass cytometry analysis results, the flow cytometry findings showed that knocking down TAZ had no dramatic effects on the number of CD4^+^ and CD8^+^ T cells in the TME of IgG isotype-treated mice; however anti-Gr-1 treatment did mildly reduce the absolute number of CD8^+^ T cell in the TME of sgCon tumors (Fig. 5C–E). In agreement with our previous mass cytometry analyses, we found significant reductions in total CD11b^+^Gr-1^+^ MDSCs, as well as the PMN-MDSC subset, between isotype-treated sgCon and sgTaz tumors; however there was no effect on the M-MDSCs. Importantly, anti-Gr-1 treatment significantly diminished total and PMN-MDSC populations within the TME of sgCon tumors but had no significant effect on MDSC abundance within sgTaz tumors, suggesting that the sgTaz TME was MDSC depleted prior to anti-Gr-1 treatment due to Taz knockdown. Indeed, there was no difference in total MDSCs or PMN-MDSCs between anti-Gr-1-treated sgCon and either isotype- or anti-Gr-1-treated sgTaz tumors, mirroring the relationships observed in tumor growth (Fig. 5F–I). Interestingly, we observed that macrophages were increased in sgTaz tumors in response to anti-Gr-1 antibody treatment, suggesting that the loss of MDSCs may also indirectly affect macrophage infiltration and/or their functionality (Fig. 5J). These findings demonstrated that tumor-intrinsic TAZ regulated mammary tumor growth at least in part via blockade of MDSC accumulation within the TME.

## Discussion

TNBC, defined by the lack of estrogen and progesterone receptors and HER2, accounts for 15–20% of all breast cancer subtypes and typically display aggressive behavior, including early recurrence and metastasis, despite intensive chemotherapy treatment [34]. Therefore, more effective therapies are urgently needed. Notably, immunotherapy has prolonged survival in various solid tumor types and represents a promising treatment strategy for TNBC. However, while several lines of evidence support the use of immunotherapy in TNBC, its efficacy has been modest and limited to a subset of cases [35]. For example, the modest clinical efficacy achieved in current clinical trials suggests that the immune suppressive TME cannot be overcome by PD-1/PD-L1 blockade alone [36]. Understanding the mechanisms that underlie these additional immune suppressive processes may open new avenues for the treatment and prevention of advanced or metastatic TNBC.

In this study, we found that reducing TAZ expression, which has long been associated with regulating cell-intrinsic mechanisms of tumorigenesis, also regulates the immunosuppressive TME, as evidenced by tumor-cell specific TAZ knockdown diminishing mammary tumor growth and metastasis in immune competent mice, but not immune deficient mice. Using RNA-seq and mass cytometry approaches, we observed several immune alterations within the TME when TAZ was deleted in the tumor cells. This reduction in tumor growth was accompanied by a reduction in MDSCs and macrophages. We further observed downregulation of the expression of certain cytokines/chemokines, such as Il33, TGF-β, Ccl5, Il1a and Cx3cl1, in the TAZ knockdown 4T1 cells. The reduction of chemokines may inhibit the recruitment of such immune suppressive myeloid populations to the TME of the TAZ knockdown tumors. Thus, future studies are warranted to explore the role of these chemokines/cytokines in detail in the mechanism of TAZ-mediated tumor progression.

It is generally accepted that PMN-MDSCs and M-MDSCs are not only phenotypically and morphologically different, but also have unique (although partially overlapping) functional and biochemical traits, which reflect their different roles under various pathological conditions [32]. Although we did not observe quantitative changes in CD8^+^ cytotoxic T cells in response to TAZ knockdown, it is important to note that we did observe significant increases in the ratio of these effectors to MDSCs. These data are consistent with the interpretation of a more immune activating or less pro-tumorigenic TME. Furthermore, using an anti-Gr-1 antibody treatment approach, we showed that treatment significantly reduced 4T1 tumor growth and PMN-MDSC accumulation within TME of control mice, but not in TAZ knockdown mice.

Most studies in the field have focused on the pro-tumorigenic roles of MDSCs on the T-cell response, which was also a focus of this study. Indeed, it has been reported that MDSCs co-localize with B-cells within the marginal zone of the spleen in tumor-bearing mice [37]. Unlike the suppressive effects of MDSCs on T cells, MDSCs may promote the proliferation and inhibit the apoptosis of B cells [38]. Interestingly, we found that anti-Gr-1 antibody treatment had no effect on the macrophage population in the control tumors, but it did increase macrophages in the TAZ knockdown tumors, suggesting that the loss of MDSCs may indirectly regulate macrophage infiltration and/or their functionality. Given the complexity of the MDSC and macrophage responses across multiple components of adaptive immunity, future studies are needed to dissect the relationship among MDSCs, macrophages, and B cell infiltration within the TME.

The recruitment of MDSCs to the TME has been demonstrated to promote tumor growth through inhibition of innate and adaptive antitumor immune functions, as well as contribute to therapeutic resistance [39]. This aligns with studies reporting that methods which reduce MDSC populations further improve the efficacy of ICIs [40]. For example, recent studies showed that using antibodies against cytotoxic-T-lymphocyte-associated protein 4 (CTLA-4) or programmed cell death 1/programmed cell death 1 ligand 1 (PD1/PD-L1) alone was insufficient to generate an effective antitumor response, however a combination of ICI with agents that inactivate MDSCs demonstrated superior efficacy against *de novo* resistance to the ICI in metastatic castration-resistant prostate cancer (mCRPC) [41].

There is emerging evidence that dysregulation of the Hippo-YAP/TAZ signaling pathway plays a critical role in the TME [42]. Several studies have shown that YAP/TAZ transcriptionally activates PD-L1 expression, thus suppressing T cell-mediated killing of tumor cells in melanoma, lung, and breast cancer [43–46]. YAP activation has been reported to drive macrophage recruitment in liver cancer models [47]. Though YAP has been found to induce MDSC recruitment in a mouse model of prostate cancer [48] and pancreatic cancer [49], how TAZ regulates immune suppressive cells in the TME of breast cancer remains largely unknown. Interestingly, Moroishi et al. found that knocking out LATS1/2, the upstream negative regulator of YAP/TAZ, inhibits tumor formation and contributes to the generation of an immune suppressive TME via secretion of nucleic-acid-rich extracellular vesicles [50]. It will be interesting to test whether those extracellular vesicles play a role in MDSC recruitment in our experimental system.

In summary, the findings of this study suggest that tumor-intrinsic TAZ expression plays a regulatory role in the accumulation of MDSCs and macrophages in the TNBC TME. The mechanisms and signaling axes involved could serve as novel therapeutic targets. Having a better understanding of TAZ-driven mammary tumor growth may support the development of innovative treatment options and improve outcomes for patients with TNBC, or other cancer types where the TAZ pathway is relevant to neoplastic progression.

## Supplementary information

Supplemental Figure and Table Legends

Figure S1

Figure S2

Figure S3

Original Western Blot

Supplemental Table 1

Supplemental Table 2

Supplemental Table 3

## Data Availability

The RNAseq data from sgCon and sgTaz tumors has been deposited to Gene Expression Omnibus (GEO) as GSE205160.
