# Supplementary material for: Loss-of-function of the hippo transducer TAZ reduces mammary tumor growth through a myeloid-derived suppressor cell-dependent mechanism
Source: Cancer Gene Ther. 2022 Jul 15;29(11):1791–800. doi: 10.1038/s41417-022-00502-0 (PMC9663307; doi:10.1038/s41417-022-00502-0)
Supplement: Supplementary file 1 — Supplemental Figure and Table Legends [file 41417_2022_502_MOESM1_ESM.docx]

**Supplemental Figure Legends:**

**Figure S1: Knockdown of Taz reduces mammary tumor growth**

1. Representative images and quantification of colony formation of 4T1 sgCon and sgTaz cells. Data are shown as the mean ± SD. Unpaired two-tailed student t-test: NS=not significant.
2. Representative images and quantification of colony formation of EMT6 sgCon and sgTaz cells. Data are shown as the mean ± SD. Unpaired two-tailed student t-test: NS=not significant.
3. Immunoblotting detection of Taz knockdown in E0771 cells. GAPDH was used as a loading control.
4. Representative images and quantification of colony formation of E0771 sgCon and sgTaz cells. Data are shown as the mean ± SD. Unpaired two-tailed student t-test: NS=not significant.
5. Immunoblotting detection of Taz knockdown in MB49 cells. GAPDH was used as a loading control.
6. Representative images and quantification of colony formation of MB49 shNT and shTaz cells. Data are shown as the mean ± SD. Unpaired two-tailed student t-test: NS=not significant.

**Figure S2: Knockdown of Taz reduces mammary tumor growth and metastasis**

1. 4T1 cells were implanted into the mammary fat pad of SCID mice; tumor growth was measured by caliper and tumor weights. Data are shown as the mean ± SD. Unpaired two-tailed student t-test: NS=not significant. n=6.
2. EMT6 cells were implanted and measured as in panel *D*. Data are shown as the mean ± SD. Unpaired two-tailed student t-test: NS=not significant. n=6.
3. Representative images and quantification of 4T1 shNT and shTaz colony formation. Data are shown as the mean ± SD. Unpaired two-tailed student t-test: NS=not significant.
4. Tumor weights were measured for 4T1 shNT and shTaz cells from SCID mice. Data are shown as the mean ± SD. Unpaired two-tailed student t-test: NS=not significant. n=6.
5. Representative images of 4T1 shNT and shTaz lung metastasis. Data are shown as the mean ± SD. Unpaired two-tailed student t-test: ***p<0.001. y-axis: met=metastasis nodules n=6.

**Figure S3: Measuring immune cell infiltration within the TME by mass cytometry analyses**

1. Representative tSNE plots of CD45, CD4, CD8 and B220 cells in 4T1 sgCon and sgTaz tumors.
2. Quantification of CD4^+^ T cells, CD8^+^ T cells and B220^+^ B cells in 4T1 sgCon and sgTaz tumors.

**Supplemental Tables**

**Table-S1 Differential expression gene changes of sgCon versus sgTaz tumors.**

**Table-S2 List CyTOF antibody panel.**
