# Supplementary material for: Loss-of-function of the hippo transducer TAZ reduces mammary tumor growth through a myeloid-derived suppressor cell-dependent mechanism
Source: Cancer Gene Ther. 2022 Jul 15;29(11):1791–800. doi: 10.1038/s41417-022-00502-0 (PMC9663307; doi:10.1038/s41417-022-00502-0)
Supplement: Supplementary file 8 — Supplemental Table 3 [file 41417_2022_502_MOESM8_ESM.docx]

**Table S3.** List of antibodies for FACS analysis

| Fluor | Antigen | Clone | Manufacturer | Catalog Number |
| --- | --- | --- | --- | --- |
| Ax594 | CD11b | M1/70 | Biolegend | 101254 |
| BV605 | F4/80 | BM8 | Biolegend | 123133 |
| Pacific Blue | Ly6G | 1A8 | Biolegend | 127611 |
| Ax700 | Ly6C | HK1.4 | Biolegend | 128023 |
| BUV395 | CD3 | 145-2C11 | BD Horizons | 563565 |
| BV785 | CD4 | RM4-5 | Biolegend | 100552 |
| BV650 | CD8 | 53-6.7 | Biolegend | 100742 |
| APC | CD45 | 30-F11 | Biolegend | 103112 |
